# Recombinant Human Bone Morphogenic Protein-2 Immobilized Fabrication of Magnesium Functionalized Injectable Hydrogels for Controlled-Delivery and Osteogenic Differentiation of Rat Bone Marrow-Derived Mesenchymal Stem Cells in Femoral Head Necrosis Repair

**DOI:** 10.3389/fcell.2021.723789

**Published:** 2021-11-25

**Authors:** Xueliang Lu, Hongyu Guo, Jiaju Li, Tianyu Sun, Mingyue Xiong

**Affiliations:** ^1^Department of Orthopedics, The First Affiliated Hospital of Henan University of Science and Technology, Luoyang, China; ^2^Clinical Medical College, Henan University of Science and Technology, Luoyang, China

**Keywords:** femoral head necrosis, cell encapsulation, injectable hydrogel, stem cells, magnesium

## Abstract

Femoral head necrosis (FHN) is a clinically progressive disease that leads to overwhelming complications without an effective therapeutic approach. In recent decades, transplantation of mesenchymal stem cells (MSCs) has played a promising role in the treatment of FHN in the initial stage; however, the success rate is still low because of unsuitable cell carriers and abridged osteogenic differentiation of the transplanted MSCs. Biopolymeric-derived hydrogels have been extensively applied as effective cell carriers and drug vesicles; they provide the most promising contributions in the fields of tissue engineering and regenerative medicine. However, the clinical potential of hydrogels may be limited because of inappropriate gelation, swelling, mechanical characteristics, toxicity in the cross-linking process, and self-healing ability. Naturally, gelated commercial hydrogels are not suitable for cell injection and infiltration because of their static network structure. In this study, we designed a novel thermogelling injectable hydrogel using natural silk fibroin-blended chitosan (CS) incorporated with magnesium (Mg) substitutes to improve physical cross-linking, stability, and cell osteogenic compatibility. The presented observations demonstrate that the developed injectable hydrogels can facilitate the controlled delivery of immobilized recombinant human bone morphogenic protein-2 (rhBMP-2) and rat bone marrow-derived MSCs (rBMSCs) with greater cell encapsulation efficiency, compatibility, and osteogenic differentiation. In addition, outcomes of *in vivo* animal studies established promising osteoinductive, bone mineral density, and bone formation rate after implantation of the injectable hydrogel scaffolds. Therefore, the developed hydrogels have great potential for clinical applications of FHN therapy.

## Introduction

Femoral head osteonecrosis (FHON) is a well-known musculoskeletal disability, which can be categorized by its treacherous inception of symptoms and mostly affects middle-aged adults (30–50 years). The osteonecrosis of femoral head leading to, leading to severe head collapse, deformity, and rapid progression to degenerative arthritis, complications that may take years (Lau et al., 2014; Wyles et al., 2015). FHON has received great attention in medical and biomaterial research because its exact treatments and medical procedures remain contentious. Protective treatments should be performed at a very early stage of FHON, and traditional clinical protocols are usually ineffective for a developed necrotic femoral head (Fang et al., 2019; Wang et al., 2020). In the past decades, numerous surgical therapies have been established for treating FHON, including rotational osteotomy, core decompression in the presence and absence of bone grafts, and electrical stimulation procedures (Kirker-Head et al., 2007). Meanwhile, the outcomes of these procedures have not been satisfactory because they help alter the weight-bearing zones of femoral head necrotic sites and only minimally influence osteogenesis. Therefore, these methods have inadequate success rates (Katiella et al., 2016). Hence, the facile strategy of biological augmentations, such as the delivery of mesenchymal stem cells (MSCs) and bone morphogenic protein-2 (BMP-2) into necrosis sites, is becoming a promising core decompression treatment that could help achieve enhanced success rates. Additionally, developed bio- and nanomaterials have been applied as alternative osteoconductive bone implants as substitutes of bone autografts and allografts, which avoids additional complications such as those caused by disease transfer and donor unavailability (Chen et al., 2020).

In recent decades, many researchers have attempted the development of potent osteogenic hydrogel implants by incorporating bioactive agents, stem cells, and osteogenic growth factors, including osteogenic proteins (OP-1/BMP-7) and bone morphogenic proteins (BMP-2; Bouyer et al., 2016; Bedair et al., 2020). BMP-2 is a well-known growth factor, which was approved by the FDA (in 2002) and is clinically recognized for use in regeneration therapies of open tibia fractures, critical-sized bone defects, lumbar spine fusions, and femoral fracture osteonecrosis. For influencing fracture regeneration, sustained release of osteogenic growth factors to the action site is required, which mainly depends on the effective immobilization and encapsulation of BMP-2 into the hydrogel scaffolds (Zhou et al., 2017; Schwieger et al., 2020). Coupling chemistry through covalent coupling and activated carboxylic acid groups would be the best choice for BMP-2 immobilization, as carboxylic groups rapidly reacts with the amine groups (lysine side chains) of proteins (Wang et al., 2012). Hence, a logical selection of injectable hydrogel components is very important for improving the therapeutic potential of hard tissue engineering and clinical regenerative medicine (Wu et al., 2019b). The development of injectable hydrogels is promising for hard tissue regeneration therapies because of their suitable-water hydrophilicity and tissue-like viscoelastic characteristics, which could provide a suitable microenvironment for the delivery of growth factors and osteoinductive stem cells (Pupkaite et al., 2019; Li et al., 2021). Specifically, three-dimensional (3D)-networked natural polymeric hydrogels can be biomimics such as extracellular matrix (ECM), which can provide high water absorption and adequate structural strength; consequently, they have been extensively applied in wound healing, bone repair, and drug delivery applications (Gyawali et al., 2013; Mahanta et al., 2017; Cheng et al., 2018; Zhang X. et al., 2020). As previously reported, prepared 3D-structured porous hydrogels have also been providing additional support for stem cell growth and proliferation because of their biological characteristics of physical support and host infiltration, and they are also capable of factor encapsulation and controlled release in tissue regeneration therapy (Liang et al., 2019). The development of injectable hydrogel networks using natural polymers such as chitosan (CS), silk fibroin (SF), and cellulose, etc. have - received considerable biomedical attention because of its well-established biodegradability *via* human enzymes and favorable *in vitro* and *in vivo* cytocompatibility with normal cell mechanisms (Cao et al., 2015; Su et al., 2016; Pankongadisak and Suwantong, 2019). As previous reported studies prepared natural polymer-based hydrogels, particularly CS and SF have attracted much in the tissue engineering and drug delivery applications. Specifically, β-glycerophosphate functionalized CS hydrogel with its thermal sensitivity has efficiently played in biomedical applications and has also prominently achieved greater regeneration of new bone tissues with combinations of other natural polymers such as SF, gelatin, collagen polymers, and inorganic components (i.e., apatite particles, bioactive glass). Nevertheless, bare CS-based gels have some shortcomings such as low strength, poor elasticity, and fast *in vivo* degradation abilities, which limit their applications in tissue regeneration applications. SF hydrogel scaffold in wet state displayed robust mechanical properties compared with other natural polymers including collagen, starch, and CS. Therefore, the combination of SF with CS polymer could be enduing them with favorable mechanical and biocompatibility properties.

CS, a cationic polysaccharide, is synthesized from chitin deacetylation and has wide therapeutic potential in biomedical applications. CS polymeric hydrogels have advantageous biological properties, including antibacterial efficiency, reduced foreign body reactions, suitable biocompatibility, and biodegradability. Furthermore, it has the ability to promote cellular proliferation, and the incorporation of specific growth factors depends on its applications (Gobin et al., 2006; Kim et al., 2010; Liu et al., 2014). SF, a natural biopolymer or/and protein regenerated from *Bombyx mori* silk, has been widely established for its orthopedic usage owing to its non-toxicity, favorable mechanical strength, lower inflammatory reactions, tunable hydrophilicity, and degradability. Specifically, the SF polymeric chain contains RGD recognition sequences (Arg-Gly-Asp), which significantly influence cell adhesion, growth, and osteogenic differentiation (Li et al., 2019; Xiao et al., 2019; Eivazzadeh-Keihan et al., 2021). Magnesium (Mg) is a well-established osteoinductive metal. It has garnered broad attention and valuable applications in orthopedic implants in bone fractures because of its role in vital physiological functional systems and its excellent mechanical strength (Young’s modulus) similar to that of natural cortical bone. Many previous reports have demonstrated that Mg-based implants significantly promote *in vivo* bone healing and regeneration *via* the assistance of the sustained release of Mg ions, which signifies that the optimal distribution of Mg in the body is a suitable host reaction (Yu et al., 2015; Laurenti et al., 2016; Lai et al., 2019).

**FIGURE 1:**
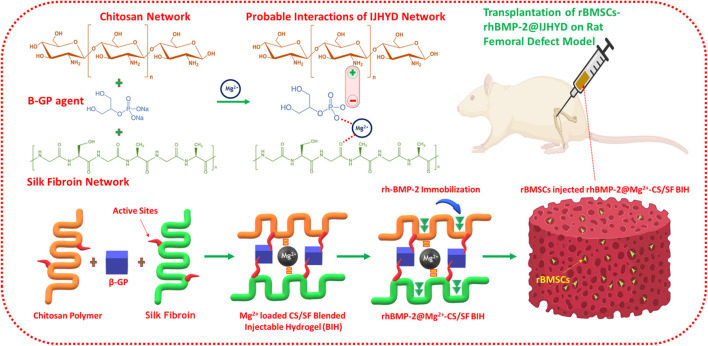
Schematic representation of magnesium (Mg) loaded chitosan (CS)/silk fibroin (SF) blended injectable hydrogel formation with probable mechanisms according to the present investigation.

In recent years, delivery and implantation of stem cells has been considered a promising and potent source for hard tissue and bone regeneration applications. This is due to their outstanding biological properties, including high cell proliferation, self-renewal ability, and differentiation of multiple cell lineages (Houdek et al., 2014; Wang et al., 2019). Moreover, stem cell types could serve as a noteworthy supporting source for hard tissue regeneration by augmenting tissue growth, metabolism, and homeostasis. From among numerous stem cell types, hematopoietic, bone marrow-derived mesenchymal, neural, retinal, and induced pluripotent stem cell types have been particularly widely applied in various clinical therapies, and they have achieved high success according to previous reports (Tan et al., 2013; Zhang D. et al., 2020; Zhu W. et al., 2020). Bone marrow-derived mesenchymal stem cells (BMSCs) play an important role in tissue engineering applications because of their good regeneration abilities; they can differentiate into regenerative cells, such as chondrocytes, adipocytes, and osteoblasts, during regenerative tissue mechanisms (Shalumon et al., 2015; Li et al., 2018; Wu et al., 2019a). In addition, BMSCs significantly and positively influence the fracture repair process, which leads to cartilaginous callus formation. Consequently, BMSCs are well known for their superior therapeutic potential and their ability to accelerate healing in fracture therapies (Gangji et al., 2011; Zhang et al., 2018). In this study, we investigated the use of a thermogelling-blended injectable hydrogel as a vehicle for multilateral delivery of BMP-2, BMSCs, and Mg ions to treat FHON. We expected that the introduction of bioactive Mg ions and growth factors into the hydrogel network could enhance osteogenesis efficiency and that BMSCs would provide bone regeneration cells. To test this hypothesis, a thermogelling hydrogel was fabricated *via* a cross-linking mechanism. This thermogelling hydrogel was a rapidly blended self-healing injectable component that permitted compatible injection into femoral defect sites, as shown in Figure 1. The gelation time, mechanical strength, pore size, morphology, and degradation were characterized. This was followed by *in vitro* biological investigations of the BMSC loading and its survival and proliferation abilities. The effects of treatment with injectable hydrogels on femoral fracture repair and regeneration ability were evaluated using rat models.

## Experimental Section

### Materials and Chemicals

Silkworm cocoons and CS (derived from crab shell; degree of deacetylation ≥95%; 600–900 cP) were obtained by Cellamatrix Co., Ltd., Hubei, PR China. Recombinant human bone morphogenic protein (rhBMP-2) was supplied by Sigma-Aldrich Aldrich (St. Louis, MO, United States). All cell culture media, including Dulbecco’s modified Eagle’s medium (DMEM), fetal bovine serum (FBS), antibiotics (penicillin and streptomycin), trypsin, basic fibroblast growth factor (bFGF), and non-essential amino acids were purchased from Servicebio Technology Co., Ltd., Hubei, PR China. Glacial acidic acid and β-glycerophosphate disodium salt (β-BG) were obtained from Sigma Aldrich (St. Louis, MO, United States). Other reagents and solutions were obtained from Sinopharm Chemical Reagent Co., Ltd., Shanghai, PR China.

### Regeneration of Silk Fibroin Solution

The stock solution of regenerated SF was prepared by degumming and dissolving, as previously reported (Singh et al., 2016; Jing et al., 2019). Briefly, raw *B. mori* silkworm cocoons were cut into small pieces and treated with Na_2_CO_3_ (0.5%) three times for degumming at 90°C for 35 min. The degummed silk fibers were washed with hot deionized (DI) water and filtered to remove sericin protein, which was attached to the surface of the SF material. The SF solution was prepared by dissolving freshly made CaCl_2_ in ethyl alcohol and DI water in a 1:2:8 molar ratio at 80°C for 5 h. The prepared colloidal solution was further dialyzed (cellulose membrane; 12,000–14,000 MWCO) for 3 days with frequent water changes at a proper duration (3 hours), which was helpful in eliminating reacted salts and impure solvents. Then, the aggregated silk fibers and impure unreacted components were removed from the SF solution by centrifugation (9,000 rpm) for 20 min. Finally, a transparent SF solution was obtained and stored at −4°C for subsequent preparation.

### Fabrication of Magnesium Incorporated Chitosan/Silk Fibroin Injectable Hydrogel

Silk fibroin-based thermogelling injectable hydrogels with a combination of CS and Mg were prepared as previously reported but with slight modifications (Pankongadisak and Suwantong, 2019; Wu et al., 2019b; Ke et al., 2020). The CS solution was prepared using 4.0 wt% CS powder dissolved in glacial acetic acid (1.0 vol%). After that, the prepared SF solution (4.0 wt%) was systematically added to the CS solution at a ratio of 1:1 in order to prepare a homogeneous solution at room temperature (32°C). To incorporate Mg into the blended hydrogel solution, 2 ml of aqueous Mg acetate [(CH_3_COO)_2_Mg] solution was gradually added to the abovementioned SF/CS solution mixture with *N*-hydroxysuccinimide (NHS)-1-ethyl-3-(3-dimethyl aminopropyl) carbodiimide (EDC) cross-linking agents under constant stirring. The newly prepared solution of β-glycerophosphate disodium salt (5 wt%) was added to the previously prepared Mg-treated blended mixture and continuously stirred at 4°C in an ice-cold bath for 30 min. The pH value was determined and maintained at an acceptable physiological neutral pH so gelation could progress. In addition, injectable CS/β-GP and CS/SF/β-GP hydrogel groups were prepared for property analysis. Finally, the prepared hydrogel samples were stored at 4°C until further use. The prepared hydrogel samples of chitosan, chitosan/silk fibroin, and magnesium loaded chitosan/silk fibroin were labeled as CS, CSSF, and Mg/CSSF, respectively.

### Rheological Evaluations of Hydrogel Groups

The gelation time of the hydrogel groups was evaluated using the vial inversion method. A gel agent was gradually added to the CS/SF mixed solution at 37°C. The gelation time was measured by observing an absence of flow after inverting the mixture solution. The gelation time measurement was repeated three times for each group to obtain accurate values. The rheological ability of each hydrogel group was determined using a TA rheometer. The storage modulus (G′) and loss modulus (G″) values were observed at temperatures between 25 and 40°C with a heating/cooling rate of 0.5°C/min at a static frequency (0.5 Hz). Viscosity measurements were performed to check the suitability of the injectable hydrogels for bone regeneration applications. Viscosity measurements were performed at shear rates of 0.1 – 400 s^–1^ at temperatures of 25 and 37°C.

### Analytical Characterizations of Hydrogel Groups

The prepared hydrogel groups were freeze dried at −40°C for 24 h and then lyophilized in a freeze dryer for further morphological, physicochemical, and mechanical characterizations. The porous morphological nature and particulate distributions in the hydrogel groups were observed and imaged under a Carl Zeiss scanning electron microscope (SEM; LEO 1450 VP; Germany) at 10 kV and a JEOL 2100F transmission electron microscope (TEM; Japan) at a specific voltage of 200 kV. The elemental distribution of the Mg-incorporated hydrogel was observed using the energy-dispersive spectroscopic (EDS) technique, which was installed using an SEM instrument setup. The purity, mesoporous order, and chemical interactions of the hydrogel groups were confirmed using x-ray diffraction (XRD) and Fourier transform infrared (FTIR) spectrometers.

### Immobilization of Recombinant Human Bone Morphogenic Protein-2 Into Magnesium-Silk Fibroin/Chitosan Hydrogel

The immobilization of rhBMP-2 (CHO-expressed-355-BM, Beijing Biolab Technology Ltd.) into the prepared scaffolds was performed as follows: the aqueous form of rhBMP-2 (1 mg/ml) was prepared by dissolving it in phosphate-buffered saline (PBS). Then, this partially dissolved suspension was kept in an ultrasonic processer and continuously stirred at 1,000 rpm for 10 min to obtain a uniformly dispersed suspension. Subsequently, the previously prepared Mg-SF/CS and SF/CS hydrogel groups were immersed in the rhBMP-2 solution and incubated for 12 h (Zhu H. et al., 2020). The rhBMP-2 immobilized Mg-CSSF was labeled as rhBM-Mg/CSSF.

### Estimation of Recombinant Human Bone Morphogenic Protein-2 Releasing Ability

The lyophilized rhBMP-2 immobilized hydrogel groups were immersed and resuspended in 10 ml of PBS buffer (pH 7.4), supplemented with sodium azide (0.02% w/v), and incubated for 24 h for primary analysis. After 24 h, the concentration of released rhBMP-2 was determined using centrifuged supernatant (2.5 ml), which was taken from the immersed medium, and the medium was refreshed with a fresh PBS buffer. The analysis was extended for another predetermined time duration (3 and 7 days), and the collected supernatants were evaluated using the BMP-2 ELISA kit (Quantikine colorimetric ELISA; DBP200).

### *In vitro* Experimental Evaluations

#### *In vitro* Isolation and Culture of Rat Bone Marrow-Derived Mesenchymal Stem Cells

Adult Sprague–Dawley rats were purchased and used to isolate rat bone marrow-derived MSCs (rBMSCs). The femurs and tibiae of the rats were systematically removed and isolated as previously described (Duan et al., 2020; Zheng et al., 2020). The isolated rBMSCs were cultured in a culture medium of DMEM supplemented with 1% penicillin–streptomycin and 10% FBS at 37°C in a culture incubator with 5% CO_2_. The culture medium was refreshed frequently at appropriate intervals, and the cultured cells were collected by treatment with a trypsin (0.25%)/EDTA (1 mM) solution. The cultured cell passages were used for further evaluation of cell survival, cell implantation, and osteogenic differentiation of the prepared hydrogel groups.

#### *In vitro* Rat Bone Marrow-Derived Mesenchymal Stem Cells Survival Analysis

Cell survival and proliferation rates were measured using the MTT assay and the Live/Dead viability kit (Invitrogen, Sweden) according to the manufacturer’s instructions. A suspension of rBMSCs (1 × 10^4^) was prepared in a cell growth medium and added to the hydrogel groups. The survival and proliferation of rat BMSCs in the hydrogel groups were monitored at 1, 3, and 7 days of culturing, and the treated hydrogel groups were moved to a fresh well plate and an MTT assay solution (100 μl) was added. The solution was allowed to incubate for 4 h, and then dimethylsulfoxide (DMSO) (100 μl) was added. Finally, the pipetted-out formazan solution was observed using a microplate absorption spectrometer at 570 nm (Gao et al., 2016; Maturavongsadit et al., 2017).

#### *In vitro* Cell Proliferations

The ability of rBMSCs to adhere and proliferate on the hydrogel groups was evaluated using confocal laser scanning microscopy (CLSM) techniques at different time intervals (1, 3, and 7 days). The rBMSCs seeded on the hydrogel groups were stained with calcein AM. Afterward, the dyed hydrogels were rinsed thrice with PBS, and paraformaldehyde (4%) was applied to immobilize the cells. Finally, the proliferation of rBMSCs was visualized by CLSM. The rBMSCs were cultured and their adhesion, and proliferation of on different hydrogel groups were observed. The culturing and dyeing protocols were the same as mentioned above. The rBMSCs that encapsulated the rhBM-Mg/CSSF hydrogel group was labeled as rBMSC@rhBM-Mg/CSSF.

### *In vivo* Experimental Investigations

All experimental procedures were performed in accordance with the approval and specifications of the International Animal Experiment Guidelines and Animal Care Committee of Henan University of Science and Technology, China. Femoral defects were created in the femoral neck canal model of adult Sprague–Dawley rats (150–200 g; 10 weeks; and *n* = 24) using a systemic surgical method. Briefly, the mice models were anesthetized using an intraperitoneal injection of ketamine and thiazine-hydrochloride at the ratios of 3.2 and 3.7 mg, respectively, per 30 g of body weight. We then created a defect at the site of the femoral neck canal opening from the superior trochanter near the lateral femoral head. The rats with the created defects (*n* = 24) were arbitrarily separated into four treatment groups postoperative: group I (*n* = 6) received rBMSCs transplanted with rhBMP-2@Mg/CSSF injectable hydrogel samples for implantation; group II (*n* = 6) received rhBMP-2@Mg/CSSF injectable hydrogel without rBMSCs for implantation; group III (*n* = 6) received Mg-incorporated CSSF injectable hydrogel without rhBMP-2 and rBMSCs for implantation; and group IV (*n* = 6) received defects without any treatment (control). X-ray radiographic images of the operated femoral defects were observed at 4 and 8 weeks postoperative. The treated animal models were systematically sacrificed 8 weeks postoperative using CO_2_ asphyxiation (Huang et al., 2020; Qayoom et al., 2020).

#### Histological Evaluations

The treated and untreated femurs were harvested, fixed in ethanol (70%), washed, and stored at 4°C. Samples were then treated with paraformaldehyde (4%) for 3 days, followed by Na-EDTA (10%) for further 7 days for the decalcification process. They were then fixed in paraffin film for histological observation at 4°C. To observe the histological morphology of the femur tissues, the samples were stained with hematoxylin and eosin (H&E) and Masson’s trichrome (MTS) staining. Finally, the stained sections were observed under a light microscope (Olympus BX 50, Japan).

### Statistical Statement

All statistical observations and data were calculated using one-way analysis of variance (ANOVA) with Bonferroni’s multiple comparison test for comparing all described analysis groups.

## Results and Discussion

**FIGURE 2:**
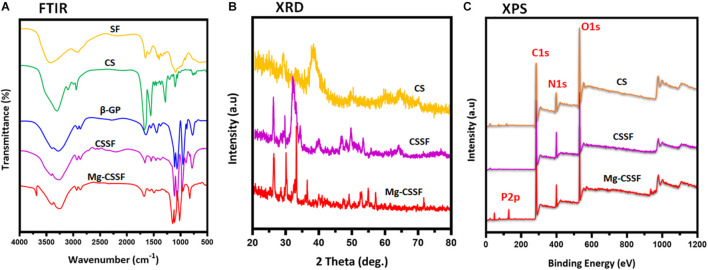
Structural interactions, chemical compositions, and elemental distributions were evaluated by spectroscopic methods of Fourier transform infrared (FTIR) **(A)**, x-ray diffraction (XRD) **(B)**, and XPS **(C)** analyses.

The design of the presented work and the probable mechanisms of material interactions are presented in the schematic illustration shown in Figure 1. Chemical structures, formation, component purity, and elemental compositions of the composite injectable hydrogels were investigated using substantial analytical methods such as Fourier Transform Infrared (FTIR), X-ray diffraction (XRD), and X-ray photoelectron spectroscopic (XPS) techniques, as shown in Figure 2. The structural interactions of the blended CS-SF biopolymeric compound with β-GP were observed in the FTIR spectra (Figure 2A), which exhibited broad and strong bands at 3,200–3,600 cm^–1^, confirming the existence of N-H and O-H stretching vibrations other than those from the intermolecular hydrogel bonds of the CS molecules. The presence of characteristic absorption bands around 880, 2,890, and 2,930 cm^–1^ is attributed to the vibration modes of the symmetric and asymmetric C-H bands, which are related to C-H out-of-plane bending and the monosaccharide ring of the CS molecule. The characteristic minor absorption band at 1,328 cm^–1^ and the strong absorption band at 1,648 cm^–1^ correspond to the stretching vibration modes of amide III (C-N) and amide I (carbonyl stretching), respectively. The presence of a sharp band at 1,060 and 1,430 cm^–1^ was ascribed to the stretching vibration of the O-C-O bridge bond and the bending vibration of the CH_2_ group, respectively, which confirms the effective blending mechanism between CS and the SF biopolymeric network. As shown in Figure 2A (CSSF), the reduced absorption band around 1,648 cm^–1^ corresponds to the overlap of amine (II) with the carbinyl bands (*C* = O) from the SF molecules, demonstrating the effectiveness of the reaction between the amine group of the CS molecule and the SF. In addition, the broad absorption band region of the CS polymer around 3,200–3,600 cm^–1^ was reduced by the addition of β-GP and SF molecules, which established strong interactions between the amine group (CS) and an effective CS/SF blend formation. The distributions of the SF molecules were confirmed by the existence of the C-N stretching (1,240 cm^–1^; amide III), N-H bending (1,530 cm^–1^; amide II), and *C* = O stretching (1,640–1,650 cm^–1^; amide I) vibration modes, which reveal random coil formations in the SF protein after composite fabrication. The noteworthy incorporation of Mg substitutes was confirmed by small characteristic absorption bands at 656 and 3,700 cm^–1^, which were attributed to the Mg-O-Mg and hydroxyl (OH) groups, respectively (Li et al., 2019).

The XRD patterns of the fabricated S hydrogel groups are shown in Figure 2B. Bare CS has a typical reflection peak in the range of 20°, implying a semicrystalline nature. The degree of crystallinity of the molecules was significantly reduced when they were blended with the SF polymeric network, which is due to the dysregulated and demolished intermolecular interactions of CS [Figure 2B (CS/SF/β-GP)]. The distribution of Mg substitutes was also confirmed by the respective diffraction peaks of the Mg matrix, which can be indexed to Mg (JCPDS No. 35-0821), as shown in Figure 2B (Han et al., 2020). In addition, the chemical composition and states of the prepared injectable hydrogels were confirmed by XPS survey spectra, as shown in Figure 2C. The characteristic peaks at 283.5, 398.2, and 531 eV correspond to the respective elemental compositions of carbon (C1s), nitrogen (N1s), and oxygen (O1s), which established the effective blending formation of CS and the SF hydrogel networks. The typical peak at 131.5 eV corresponds to P2p, implying a substantial influence of β-GP on the thermal gelling of the CSSF injectable hydrogel (Chen et al., 2012; Deng et al., 2017). The XPS results of the Mg-CSSF hydrogel demonstrate successful formation of blended gel by electrostatic interactions due to the oppositely charged networks of CS and SF, as described in Figure 1.

**FIGURE 3:**
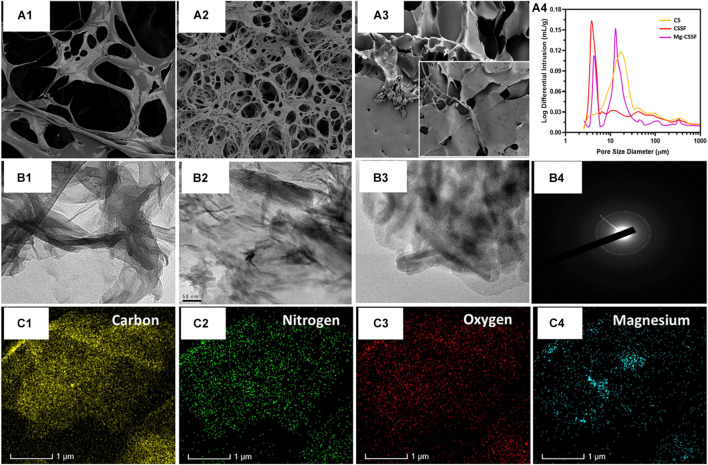
Morphological structure, porous network, and component distributions were investigated and presented using scanning electron microscope (SEM) and transmission electron microscope (TEM) microscopic instruments and EDX mapping results; **(A^1–3^)** are the SEM observations of the CS, magnesium loaded chitosan/silk fibroin (Mg/CSSF) and, Mg/CSSF hydrogel groups, respectively, which exhibit improved porous morphology consistent with the pore size analysis results **(A^4^)**. Transmission microscopic observations of hydrogel groups are displayed as **(B^1^)** CS, **(B^2^)** Mg/CSSF, **(B^3^)** Mg/CSSF, and **(B^4^)** SAED patterns of Mg/CSSF. **(C)** Elemental distributions of C, O, N, and Mg elements were confirmed by EDX mapping analysis.

The porous morphology and interconnected structure of the lyophilized hydrogels were observed and visualized by SEM analysis, as shown in Figures 3A^1–3^). In particular, the blended CS/SF/β-GP hydrogel group exhibited a microporous morphology with a well-arranged network structure. The reaction with SF polymers provides more active cross-linking sites and an opaque network with CS molecules with the assistance of β-glycerophosphate molecules, which also leads to the effective thermal gelling ability of the injectable hydrogel (Zhou et al., 2019). The Mg ion-incorporated hydrogel had a narrower distribution and interconnected morphological structure, which exhibited special shaped Mg particles attached to the porous hydrogel surface (Figure 3A^3^). The average pore size distributions of the CS/β-GP, CS/SF/β-GP, and Mg- CS/SF/β-GP injectable hydrogels were 45, 30, and 25 μm (Figure 3A^4^), respectively, demonstrating the well-connected and reduced pore size of the blended CS and SF copolymeric networks with the addition of a β-GP thermogelling substrate. Normally, the favorable porous nature of the hydrogel groups used in tissue engineering applications is very important for their affinity to solvents, such as body fluids and safe-sided secured cavities, for cell injection and growth. The layered structures of the hydrogel groups were further confirmed by TEM analysis, which revealed the blending formation of CS and SF polymeric networks with the assistance of β-GP, as displayed in Figures 3B^1,2^. The distribution of spherically shaped Mg substitutes in the hydrogel network was also confirmed by TEM microscopy and SAED patterns, as shown in Figure 3B^3,4^, respectively. The elemental compositions of the β-GP-assisted Mg-CS/SF hydrogel and other hydrogel groups were determined by EDX mapping analysis (Figure 3C), with which a uniform dispersal of carbon, oxygen, nitrogen, and Mg in the hydrogel network was determined. The structural and morphological characteristics of the prepared blended Mg-CS/SF hydrogel group would prominently maintain their structural stability and porous structure, which makes them effective carriers for cells and growth factors in tissue engineering applications. As a bone implant material, in addition to favorable cytocompatibility, it also needs improved mechanical abilities for strong regeneration treatments. Unfortunately, the mechanical properties of natural polymers should be improved with compositions of blending other polymers and incorporation of suitable inorganic components. In the present investigations, we have achieved enhanced structural and mechanical properties of CS compound with SF and Mg ions with assistance of thermal-sensitive β-glycerophosphate. To the best of our knowledge, SF and CS materials are the most abundant biopolymers available in the world, which displayed outstanding biocompatibility for cell attachment and growth. Hence, we have demonstrated blending SF/CS bio polymeric hydrogel for the potential bone regeneration applications.

**FIGURE 4:**
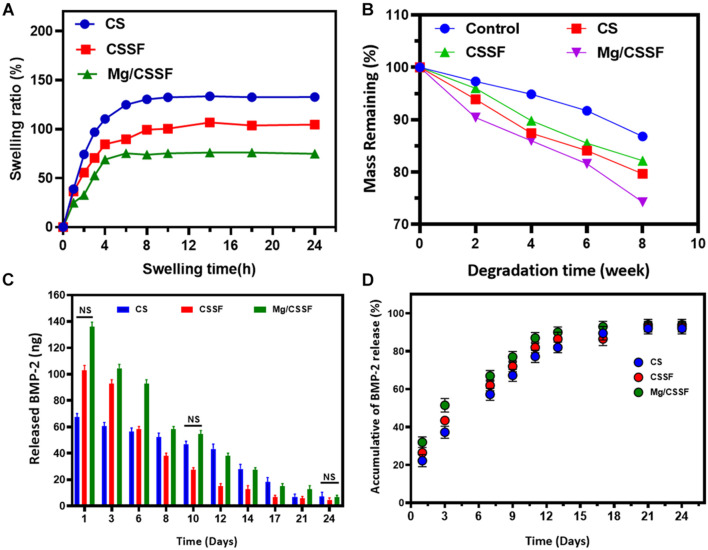
Swelling ratio **(A)**, biodegradation behaviors **(B)**, and *in vitro* recombinant human bone morphogenic protein-2 (rhBMP-2) release profiles **(C,D)** of fabricated injectable hydrogel groups [CS, chitosan/silk fibroin (CSSF), and Mg/CSSF] for different time durations, as required for experimental accuracy.

Investing the swelling capabilities of the hydrogel groups is vital for preparing them for tissue regeneration applications because it primarily influences the transfer of oxygen and nutrients to the cell compatibility. The swelling profiles of the fabricated hydrogel groups were studied using PBS at appropriate biological conditions (pH 7.4, 37°C) and are presented in Figure 4A. The results show that the formed CS gel has a very high swelling ability compared with other composite hydrogel groups because of its greater hydrophilic nature, whereas CS molecules substituted into the SF network established a controlled swelling percentage, which significantly improved with increasing time. Additionally, the swelling ability was reduced with an increasing percentage of Mg substrates, demonstrating a significant enhancement of the hydrophobic character with substitution. This is consistent with the results of the water contact angle data, as shown in Supplementary Figure 1. Observation of the water uptake ability results showed that the water uptake ability of Mg-CSSF hydrogel group significantly decreased from 92.9 ± 1.2 to 77.5 ± 2.3% with increasing time between 0 and 8 h. These outcomes could be attributed to the influence of ethanol treatment on the SF polymeric network, which led to the effective formation of β-sheets in the hydrogel network. Consequently, during ethanol treatment, the hydrophobic regions of alanine and glycine in the SF protein greatly contributed to the formation of more β-sheet structures. This demonstrated the increased hydrophobicity of the hydrogels with a higher number of β-sheet networks (Liu et al., 2020). The favorable and suitable bio-degradation properties for implantable bio-materials are highly vital to apply in the field of tissue engineering applications. An *in vitro* degradation analysis was performed in a PBS medium by determining the weight loss of the lyophilized hydrogel groups, as shown in Figure 4B. The remaining masses of the prepared SF (control), CS, CSSF, and Mg/CSSF hydrogel groups were 86.8, 79.66, 82.17 ± 1.2, and 74.75 ± 2.5%, respectively, after incubation for 8 weeks. The remaining mass of CSSF was larger than that of CS, indicating that blending of SF with CS molecules affects its biodegradation properties, because the β-sheet structure of the SF network leads to slower degradation. Furthermore, the incorporation of Mg substitutes influenced the biodegradation of the hydrogel groups; they exhibited accelerated degradation after 6 weeks. Generally, when the degradation abilities of implantable materials are too fast or too slow, they may result in reduced regeneration efficiency and cell growth due to frequent mechanical loading. The release kinetics of immobilized rhBMP-2 from the hydrogel groups were evaluated, and a cumulative BMP-2 release was revealed, as shown in Figures 4C,D. The results demonstrate that the Mg/CSSF and CSSF blended hydrogel group caused a burst release in the beginning and then provided controlled release with increasing incubation time. The release kinetics of the hydrogel groups are highly consistent with those reported in previous studies, which confirms that the release efficiency is sufficient for achieving better outcomes in bone regeneration therapy (Nageeb et al., 2012).

**FIGURE 5:**
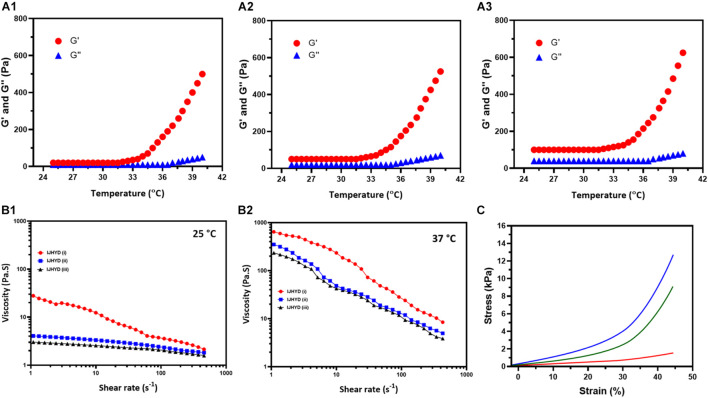
Systematic observations of temperature dependent G′ and G′′ functions for the **(A^1^)** CS, **(A^2^)** Mg/CSSF, and **(A^3^)** Mg/CSSF hydrogel groups. **(B^1,2^)** Viscosity vs. shear rate observations of prepared hydrogel groups at 25 and 37°C, respectively. **(C)** Stress–strain curve results of the hydrogel groups.

The thermal-based gelation of the CS/SF blended polymeric network with the assistance of β-GP substrates was investigated using phase diagram analysis (Temp. vs. Conc. of Gelation) and is shown in Figure 5; the results imply that the blended polymeric network has a large gelation window when compared with that of bare CS gel. Furthermore, the addition of the SF polymeric group led to greater control of the gelation window, indicating that inter- and intramolecular hydrogen bonding interactions are greatly influenced by the main factor of the gelation mechanism in the blended polymeric network, as shown in Figures 5A^1–3^ (CS, CSSF, and Mg/CSSF). A favorable gelation viscosity is a very important factor for biomedical applications of injectable hydrogels. In the current study, the viscosities of the prepared hydrogel groups were modified with shear rates at different temperatures (25 and 37°C), as shown in Figures 5B^1,2^. The viscosity of all hydrogel groups decreased gradually with increasing shear rate at 25 and 37°C. Nonetheless, viscosity changes in the hydrogel groups were superior at 25°C when compared with those at 37°C. The storage modulus (G′) of the CSSF and Mg/CSSF groups increased significantly at the transition temperature (37°) of gelation when compared with that of the CS group, which revealed that CSSF/β-GP has a great gelation ability; it changes the solution to a gel, as shown Figure 5C. A higher storage modulus (G′) was observed for the blended hydrogel groups compared with CS/β-GP; this confirms the formation of effective hydrogen bonds between NH^3+^ (CS) and −OH (SF) with the respective phosphate groups (−PO_4_^3–^) in the β-GP molecule (Zhou et al., 2015; Dang et al., 2017). The presented results demonstrate that the established hydrogel groups significantly influenced the shear-thinning behavior, which revealed that the injectable hydrogel could be appropriate for injection into a bone regeneration animal model. Importantly, the stiffness of the prepared hydrogel groups has been investigated and presented by dynamic mechanical analysis (DMA) method. The Young’s modulus of hydrogel groups was observed for tan delta at equilibrium swollen state as shown Supplementary Figure 4. The results exhibited that Young’s modulus values have been enhanced for the Mg incorporated CSSF when compared to the other hydrogel groups, which confirm that distribution of Mg ions would increase stiffness and elastic properties of the hydrogel product. As previously reported, stress transfer between polymer and inorganic components has been increased when preparing composite structure due to influences of cooperative hydrogen-bonding network. Meanwhile, the mechanical property differences between bare SF and blending CSSF hydrogel groups corresponded to the interface zone between blending polymeric groups, prominently the hydrogen bonding ability of the intercalating species, which enhances the mechanical properties of the hydrogel. Based on the mechanical analyses, it was concluded that blending CSSF polymeric hydrogel chain has played a greater role in stiffness and other mechanical strength. The blending of negatively charged CS with β-glycerophosphate would be prominently influencing the mechanical improvement due to its electrostatic interaction with SF polymeric chain. Frequently, the prepared blending hydrogel with incorporation of Mg showed favorable mechanical ability to meet the requirement of femoral head necrosis (FHN). Generally, hydrogels from natural polymers are potential implant materials for bone tissue engineering due to its suitable mechanical stability and biocompatibility to normal cells. In the present investigations, bioactive Mg and morphogenic proteins were incorporated to improve the cell adhesion behaviors and regeneration ability. Recently, blending hydrogels with CS and SF polymers can effectively promote cell adhesions as well integrate with host tissues, which have been extensively applied in bone regeneration applications. Importantly, the blending polymeric networks creating elastic and high modulus network with addition of Mg ions could substantially improve tissue adhesiveness and cell affinity with normal fibroblast and stem cells.

**FIGURE 6:**
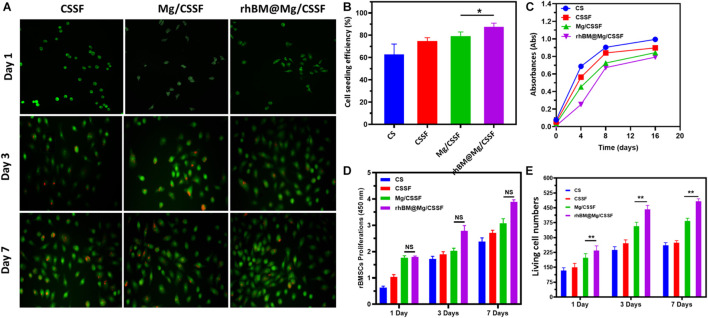
**(A)** Live/Dead cell morphology and proliferations of the rat bone marrow-derived MSCs (rBMSCs) cultured with prepared hydrogel groups observed under confocal laser scanning microscopy (CLSM) at different incubation times (1, 3, and 7 days). **(B)** Cell seeding efficiency, **(C)** cell survival absorbance, **(D)** proliferation rate, and **(E)** live cell number of rBMSCs encapsulated in hydrogel groups for different incubation durations. ****p < 0.0001; ***p = 0.0001–0.001; **p = 0.001–0.01; *p = 0.01–0.05; *p* ≥ 0.05 means not significant (NS).

The present study was developed for the delivery of rBMSCs into the FHN site using biocompatible injectable hydrogel scaffolds as an efficient vehicle, as schematically illustrated in Figure 1. Hence, investigation of the cell cytocompatibility, proliferation rate, and survival rate of the hydrogel groups was necessary for progress in this study. According to *in vitro* evaluations with a cell counting kit-8 (CCK-8) assay, the cell survival rates of the rBMSCs were not affected by the prepared hydrogel groups (Figure 6C), which reveals that the compositions of Mg-loaded blended hydrogel groups at optimal concentrations have greater cell compatibility. The isolated rBMSCs from the SD rats were systematically encapsulated in the prepared hydrogel groups, as schematically shown in Figure 1. After that, rBMSC-loaded hydrogel groups were seeded in a cell culture medium (DMEM) showing that the encapsulated cells had a good seeding efficiency (Figure 6B), greater proliferation rate (Figure 6D) and enhanced live cell numbers (Figure 6E), as greatly consisted with visualization by confocal (CLSM) microscopy. The live/dead cells were observed using dual staining, such as Calcein AM (green color) and EthD-1 (red color), as displayed in Figure 6A. The fluorescence images demonstrated the improved elongated and flattened morphology of the rBMSCs with increasing number of postencapsulation days (3 and 7), which revealed that hydrogel vehicles allow for homogeneous cell distribution and proliferation and provide good cell survival ability (>90%) for different culture durations. Furthermore, cluster-like formations of rBMSCs were observed in all hydrogel groups at 7 days postencapsulation, demonstrating that the hydrogels offer appropriate compatibility and that the gelation mechanism of β-GP does not disturb cell viability after encapsulation. The proliferation rate and living cell number of the rBMSCs loaded in the blended hydrogel groups were further evaluated using the CCK-8 assay, as shown in Figure 6D. The observations indicated that the cell numbers greatly increased during days 3 and 7 of the culture, and significant differences were observed when the number of postencapsulation days increased. Based on these observations of *in vitro* cell compatibility analyses, we conclude that the prepared hydrogel groups provide strong biological support for adequate nutrient and oxygen transport due to their fibrillar walls and evenly distributed porous network. This indicates that rBMSCs could easily attach to the wall and proliferate efficiently in hydrogel vesicles.

**FIGURE 7:**
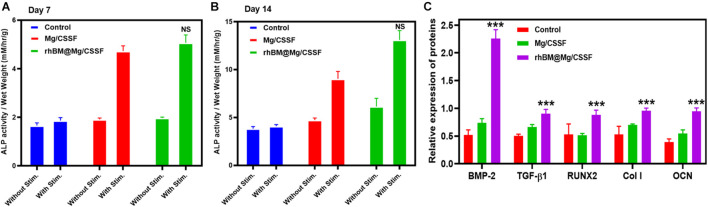
Analysis of osteogenic differentiation efficacy of rBMSCs on injectable hydrogel groups. **(A,B)** Alkaline phosphatase (ALP) activity at different incubation times of 7 and 14 days, respectively. **(C)** Quantitative analysis of osteogenic gene expressions of rBMSCs seeded on hydrogel groups at day 14 of the culture period (*n* = 3, *p < 0.05, ***p value 0.0001–0.001 of significant differences).

The osteogenic differentiation potential of rBMSCs in the developed hydrogel groups is very important for bone implantation and regeneration therapies. The osteogenic potential of the rBMSC-embedded hydrogel groups was investigated using biochemical analysis and Reverse transcription Polymerase chain reaction (RT-PCR). Alkaline phosphatase (ALP) is a potential Ca^2+^ carrier, which is a well-known early marker of osteogenic differentiation in BMSCs (Shang et al., 2019). Hence, the osteoinductive ability of the hydrogel groups was studied in two kinds of culture media: one in the presence of an osteoinductive stimulator and the other in its absence. The observations of the rBMSC@rhBM-Mg/CSSF group indicated greater ALP upregulation activity when compared with the other hydrogel groups at days 7 and 14 of the cultures, as shown in Figures 7A,B, respectively, which confirms that the prepared hydrogel can enhance bone regeneration. In addition, osteogenic differentiation was further confirmed by bone-specific extracellular proteins, including BMP-2, TGF-β1, RUNX2, Col1, and OCN. The mRNA expressions level in the rhBM-Mg/CSSF group was significantly enhanced compared with the other hydrogel groups after 7 and 14 days of culture, respectively (Figure 7C), indicating that the developed blended hydrogel groups with rBMSCs have favorable influences and positive factors for the effective bone formation.

**FIGURE 8:**
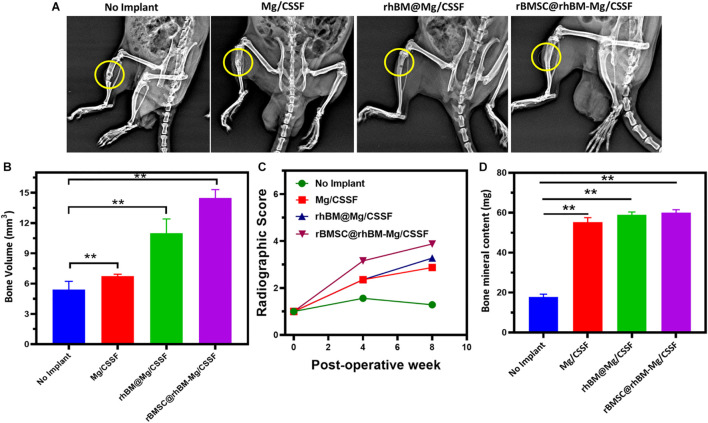
**(A)** Radiography (x-ray) observation of femoral defects in RD rat models transplanted with rBMSCs encapsulated rBMSC@rhBM-Mg/CSSF and other comparative hydrogel groups (no implant defect models considered as control). Quantitative investigations of **(B)** bone volume (mm^3^), **(C)** radiographic score, and **(D)** bone mineral content (BMC) on hydrogel treated *in vivo* models. **p = 0.001–0.01.

**FIGURE 9:**
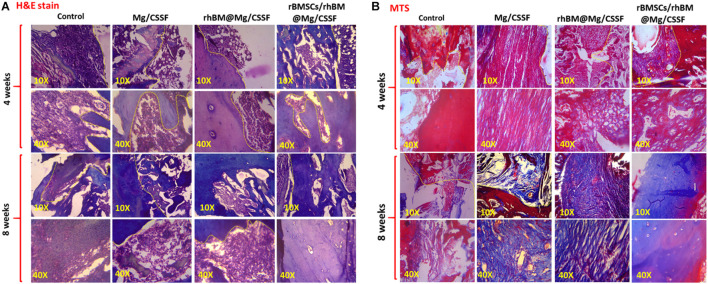
Histological sections of treated femoral defect models implanted with injectable hydrogel groups were observed by using hematoxylin and eosin (H&E) **(A)** and Masson’s trichrome (MTS) stains **(B)**. The histological microscopic observations were observed in two magnifications (10× and 40×) to show bone remodeling and regeneration.

The *in vivo* treatment efficiency of the rBMSC-encapsulated injectable hydrogels was investigated and presented using x-ray photographs and histological analyses, as shown in Figures 8, 9. X-ray observations of the rat model tibia bone group treated with rBMSCs/rhBM-Mg/CSSF showed excellent bone remodeling and regeneration ability with parent cortices after 8 weeks postimplantation when compared with the other hydrogel groups and the control (Figure 8A). In addition, the Mg/CSSF and rhBM-Mg/CSSF hydrogel groups exhibited greater sponge-like bone regeneration at the defect site. This can be confirmed by the complete bridging callus formation of the necrotic bone. Clinically, bone implantation treatment using fixation devices has many complications, including non-union of the fracture site and implant loosening, which leads to implant failure. In the present investigation, the combined effect of rBMSCs transplantation and sustainable release of rhBMP-2 through the developed hydrogel groups in an appropriate manner could induce more callus formation and bridge defect sites much more rapidly than in the control groups. The x-ray findings demonstrate that the rBMSC-encapsulated hydrogel groups provided early osteogenesis support and strength to the subchondral bone to prevent wrapping bone collapse. The bone healing ability of the hydrogel groups was further established by the quantitative outcomes of bone volume density, radiographic score, and bone mineral content. The rBMSCs and rhBMP-2-loaded hydrogel group had superior effects on BV/TV (Figure 8B), radiographic score (Figure 8C), and BMD (Figure 8D) when compared with the other groups, which demonstrates that they may have greatly contributed to new bone formation.

The morphology of the newly formed bone was microscopically observed by histological evaluations using H&E and MTS stains using harvested and decalcified femoral models treated with injectable hydrogel, as shown in Figure 9. The histological results demonstrated that the developed hydrogel groups had induced substantial filling and mineralization in the defect sites at 4 and 8 weeks postimplantation compared with other groups. In addition, the rhBM-Mg/CSSF and Mg/CSSF groups also exhibited significant ability to cause regeneration and new bone formation, which is confirmed by the fibrous connective tissues filled between the defect sites in end-to-end soft tissues, as shown in the H&E staining images (Figure 9A). Meanwhile, the histology of the defect site without implantation did not exhibit any noticeable bone regeneration in the engineered tissue at 4 weeks postimplantation. The histology of the cell-encapsulated hydrogel group showed a greater extent of new bone area formation and could also clearly illustrate the differentiation of rBMSCs in MTS staining images. In addition, the histological images displayed aggregates of red marrow and regenerated blood vessels in the rhBMP-2@rBMSCs-loaded Mg/CSSF groups, indicating the occurrence of effective vascularization and medullary cavity formation in the newly generated bone tissues. Notably, the bridged femoral defect site had noticeably improved after gel implantation, as shown by MTS histology (Figure 9B), signifying that the implanted hydrogel groups had promising effects for improving the mineralization of newly formed collagen fiber. Generally, the process of vasculature provides essential nutrients for bone osteogenesis mechanism and eliminates waste products, which are essentially contributing to bone regeneration. As previously reported, incorporation of growth factors and morphogenic proteins (BMP-2) into the porous scaffolding materials has been exhibited to indorse angiogenesis and osteogenesis during *in vivo* treatments. Hence, the sustained delivery of growth factors and stem cells would be an effective treatment strategy to improve osteogenesis and angiogenesis for FHN regeneration therapy.

**FIGURE 10:**
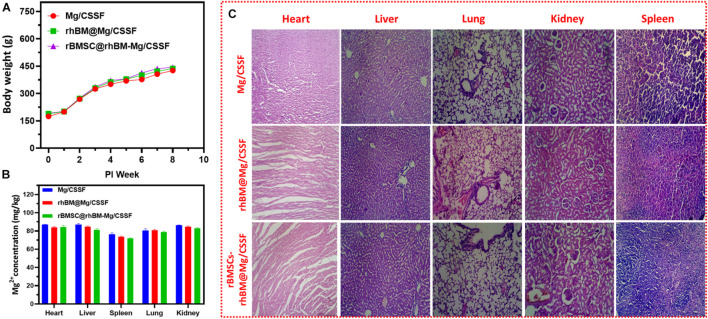
*In vivo* biosafety assessments: **(A)** body weight changes of RD rats after implantation of hydrogel groups according to postimplantation time (weeks). **(B)** Quantitative observations of average value of Mg^2+^ ions concentrations in the different organs of treated models after 8 weeks and **(C)** histological observations of treated models’ microstructures of heart, liver, lung, kidneys, and spleen with H&E staining.

The toxicity effects of the implanted hydrogel groups were evaluated using experimental animal models for 8 weeks postimplantation (Figure 10A). The results demonstrated that no significant differences were observed between the body weights of the treated animals and those in the control group. Additionally, H&E-stained sections of hearts, livers, spleens, lungs, and kidneys harvested from the animals treated with different hydrogel groups did not exhibit any noteworthy changes (Figure 10C). The Mg^2+^ ion concentration presented in the animal organs was evaluated and presented in Figure 10B, which exhibits the optimal Mg^2+^ ions distributed and would be more favorable osteogenesis in the FHN regeneration treatment as previously reported (Zhao et al., 2016; Jia et al., 2017). Additionally, blood electrolytes, liver function, and renal function of the experimental animals were investigated for *in vivo* compatibility with the hydrogel groups (Supplementary Figure 3). Those were shown no statistical changes in blood Mg ion concentrations, liver functions, and blood cell counts for the different hydrogel groups as shown in Supplementary Figure 3. These observations demonstrate the importance of gel composition, pore size, and incorporation of nanosubstitutes in cell-encapsulated injectable hydrogel groups for femoral head regeneration therapy. In previous reports, incorporation of some specific biological elements (e.g., Mg, Ca, and Zn) provides the greater bone regeneration and biomedical properties in the tissue engineering applications. In addition, Mg^2+^ ions are abundant in normal cells and very important for cellular functions. The incorporation of Mg^2+^ into the implant materials could influence numerous biological mechanisms including DNA stabilization, enzyme activation, ion channels regulations, cell growth, and proliferations with regulating metabolisms. Prominently, Mg^2+^ ions can indorse the integrin expressions of human osteoblast integrins and adhesion behaviors in the bone repair processes. Notably, Mg-loaded CS/SF blended hydrogel groups displayed a favorable microenvironment for cell growth, proliferation, and compatibility owing to their unique properties of porous networks and smooth surfaces. Additionally, Mg substitutes are evenly distributed in blended hydrogel networks, which could facilitate cell movement and transport of cell metabolites, oxygen, and nutrients, which are required for cell growth within the hydrogel network (Sun et al., 2017). The introduction of Mg into the scaffolding materials would be an effective alternative to meet a clinical need in the bone implantations, which are a facile and effective material in the tissue engineering applications. Importantly, incorporation of appropriate concentrations of Mg components in the hydrogel scaffolds serve as potential candidate for bone regeneration applications due to their favorable biocompatibility, osteoinductive, degradable behavior, and elastic modulus, which are more like natural bone compounds. Additionally, Mg-incorporated hydrogel scaffolds could meet the standard of smart implantation materials that can routinely degrade and disappear after completion of their role in the human body. The prepared blended injectable hydrogel material has significant advantages in clinical procedures: such as simple operative method, creation of small-sized wound infliction, shorter operation time, and, importantly, identically matching shape and structures of injectable hydrogels to femoral head defect area. Recently, surgeons have considered augmentation protocol combination with core compression methods, such as scaffolds preparation using suitable polymeric components with growth factors, morphogenic proteins (BMP), and stem cells, to improve bone regeneration in the femoral head. At the same time, there are limited studies done on bone regeneration ability and neovascularization of blending hydrogel materials for the FHN treatments. The BMP-2 has an exceptionally short half-life in tissue engineering applications when compared to the complete osteogenic response. In previous clinical experiments, favorable, and optimal doses of BMP-2 were required for successful improvement of bone regeneration and avoid adverse side effects. Hence, the main objective of the present investigation was sustained release of BMP-2 molecule from the suitable carriers as well improve cellular activity to enhance potential osteonecrotic bone regeneration and substitution in FHN treatment.

Generally, the effectiveness of bone regeneration is studied by the interactions between prepared implantable hydrogel groups and cells in the defected area. Once, the hydrogel materials are implanted into the defect site, the biological immune system will recognize the antigenic determinant presented in the composited hydrogel; also, the immune system may possibly discard the implant materials. Immunological rejection has played a main role in implant materials in bone regeneration treatment. Hence, the main achievements of bone implant materials are suitable biocompatibility and reducing immunological rejection. In our observed results, biocompatibility assay proved outstanding BMSC cell compatibility with the prepared hydrogel groups. In addition, osteogenic markers and ALP staining also confirmed that blended hydrogel can support BMSC cell activity as well promote osteogenic differentiation *in vitro* and *in vivo*. The combined actions of rBMSCs and rhBMP-2 have promising effects on tissue regeneration mechanisms sustainably delivered from injectable hydrogel groups, which could prove to be highly beneficial in future investigations focused on femoral head regeneration therapy.

## Conclusion

In summary, we reported the fabrication of a novel thermogelling injectable hydrogel vehicle with an SF/CS-blended biopolymeric composition for effective cell delivery for femoral head regeneration therapy. The incorporation of the β-GP thermogelling agent into the blended solution can significantly improve the temperature-dependent gelation with strong intermolecular interactions between the SF and CS molecules. More importantly, in order to fabricate a suitable cell delivery vehicle for tissue regeneration applications, Mg^2+^-incorporated hydrogels were successfully prepared with a suitable cell microenvironment porous structure. The developed biomaterials had beneficial properties for tissue repair applications, including evenly distributed porous morphology, thermogelling, and improved *in vitro* cell growth, proliferation, and angiogenesis. *In vivo* animal experiments illustrated that the injected rBMSCs-rhBMP-2-loaded hydrogel groups could significantly improve the bridging callus formation of the necrotic bone and effectively improve the BV and BMD. Therefore, we hypothesize that the Mg^2+^ and rhBMP-2-incorporated injectable hydrogel encapsulated with rBMSCs has a potential application in femoral head regeneration as well as other hard tissue engineering applications.

## Data Availability Statement

The original contributions presented in the study are included in the article/Supplementary Material, further inquiries can be directed to the corresponding author.

## Ethics Statement

The animal study was reviewed and approved by the all experimental procedures were performed in accordance with the approval and specifications of the International Animal Experiment Guidelines and the Animal Care Committee of Henan University of Science and Technology, China.

## Author Contributions

XL, HG, JL, and TS supported with synthesis, characterization, molecular, and biochemical analysis, data curation, formal analysis, and validation. MX helped with supervised the research. All authors contributed to the article and approved the submitted version.

## Conflict of Interest

The authors declare that the research was conducted in the absence of any commercial or financial relationships that could be construed as a potential conflict of interest.

## Publisher’s Note

All claims expressed in this article are solely those of the authors and do not necessarily represent those of their affiliated organizations, or those of the publisher, the editors and the reviewers. Any product that may be evaluated in this article, or claim that may be made by its manufacturer, is not guaranteed or endorsed by the publisher.
