# Supplementary material for: Recombinant Human Bone Morphogenic Protein-2 Immobilized Fabrication of Magnesium Functionalized Injectable Hydrogels for Controlled-Delivery and Osteogenic Differentiation of Rat Bone Marrow-Derived Mesenchymal Stem Cells in Femoral Head Necrosis Repair
Source: Front Cell Dev Biol. 2021 Nov 25;9:723789. doi: 10.3389/fcell.2021.723789 (PMC8656218; doi:10.3389/fcell.2021.723789)
Supplement: Supplementary file 1 [file Data_Sheet_1.docx]

**Supplementary Information’s**

**Recombinant human BMP-2 immobilized fabrication of Magnesium functionalized Injectable hydrogel to controlled-delivery and osteogenic differentiation of rBMSCs in Femoral Head Necrosis repair**

**Xueliang Lu^a^, Hongyu Guo^b^, Jiaju Li^b^, Tianyu Sun^b^,** **Mingyue Xiong*^a^**

^a^Department of orthopedics, the First Affiliated Hospital of Henan University of science and technology, Luoyang 471000, PR China.

^b^Clinical Medical College, Henan University of Science and Technology, Luoyang 471003, PR China.


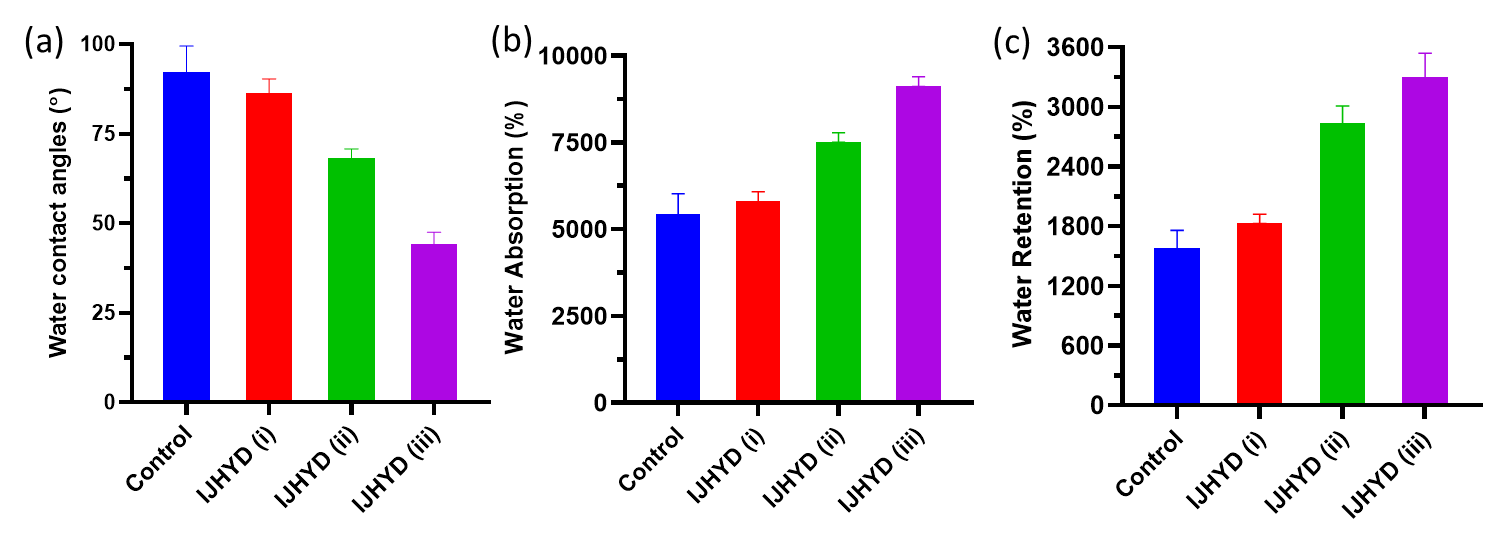


**Fig. SI 1.** Quantitative analysis of water contact angle (a), water absorption percentage (%) and (c) water retention properties of prepared hydrogel groups.


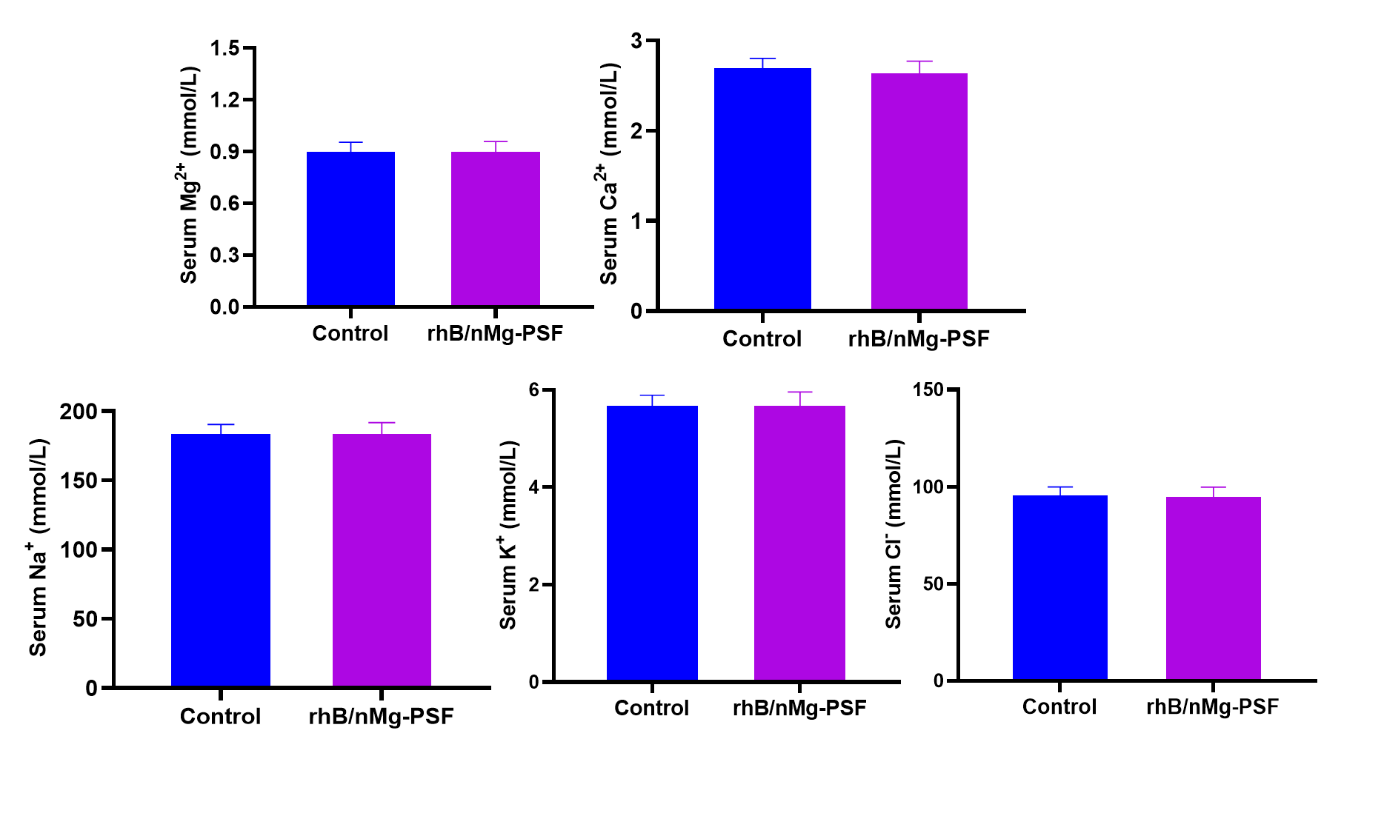


**Fig. SI 2.** Quantitative observation of average concentration of different ions presented on the blood of treated animal models after 8 weeks of implantations.

**Fig. SI 3.** Young’s modulus observation of the prepared hydrogel groups (Control (CS), CSSF, Mg/CSSF to know the stiffness behaviour.


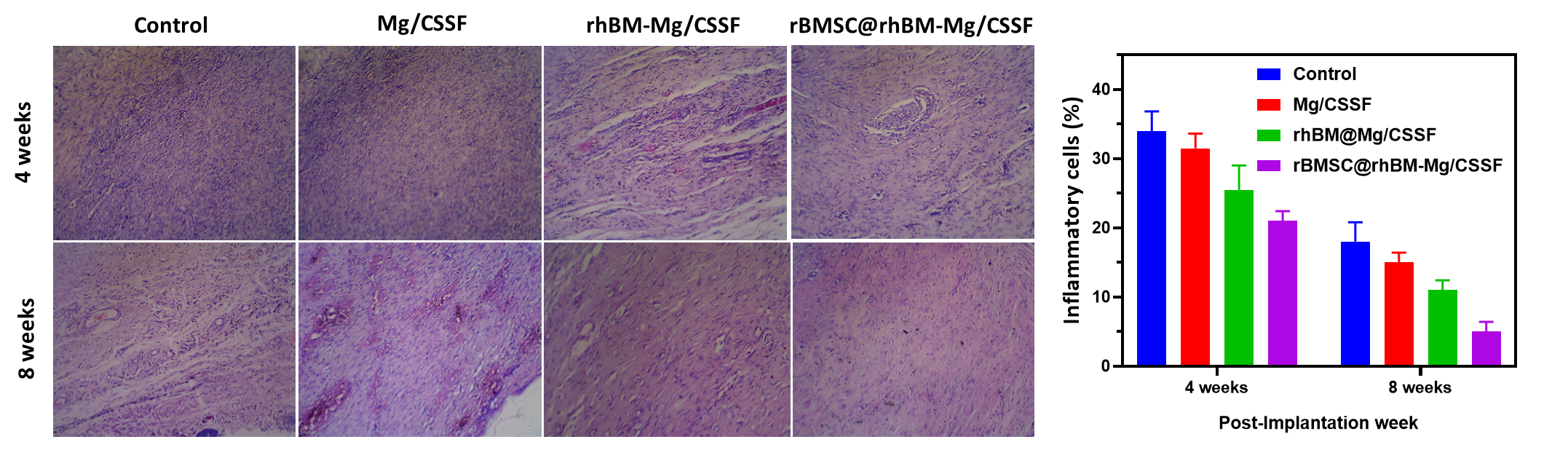


**Fig. SI 4.** Histological observations of surrounding skin tissues of implanted site for different treatment materials and quantitative measurements of inflammatory cells (%) for 4 and 8-weeks of post implantation.
